# The unique role of innate lymphoid cells in cancer and the hepatic microenvironment

**DOI:** 10.1038/s41423-022-00901-1

**Published:** 2022-08-12

**Authors:** Sophie Curio, Gabrielle T. Belz

**Affiliations:** The University of Queensland Diamantina Institute, Faculty of Medicine, The University of Queensland, Woolloongabba, QLD 4102 Australia

**Keywords:** Inflammation, Tumor, ILC subsets, Cytokines, Progenitors, Innate lymphoid cells, Tumour immunology, Chronic inflammation

## Abstract

Cancer is a complex disease, and despite incredible progress over the last decade, it remains the leading cause of death worldwide. Liver cancers, including hepatocellular carcinoma (HCC), and liver metastases are distinct from other cancers in that they typically emerge as a consequence of long-term low-grade inflammation. Understanding the mechanisms that underpin inflammation-driven tissue remodeling of the hepatic immune environment is likely to provide new insights into much needed treatments for this devastating disease. Group 1 innate lymphoid cells (ILCs), which include natural killer (NK) cells and ILC1s, are particularly enriched in the liver and thought to contribute to the pathogenesis of a number of liver diseases, including cancer. NK cells are an attractive, but underexplored, therapeutic target in hepatic disease due to their role in immunosurveillance and their ability to recognize and eliminate malignant cells. ILC1s are closely related to and share many phenotypic features with NK cells but are less well studied. Thus, their utility in immunotherapeutic approaches is not yet well understood. Here, we review our current understanding of ILCs in cancer with a particular focus on liver and liver-related diseases.

## Introduction

Every year, an estimated 19.3 million new cases of cancer are diagnosed, and approximately 10 million deaths occur worldwide [1, 2]. Immunotherapy has had a major impact in harnessing the body’s own immune system to fight cancers, including hepatic cancers [3]. Regardless, only approximately 20% of patients demonstrate durable responses to immunotherapy, leaving the majority of patients vulnerable to ongoing progressive disease and often with few therapeutic options [4]. This highlights a major gap in our knowledge and that we still have much to learn about the intricacies of designing highly effective therapies.

Environmental factors such as a Western diet, smoking and consumption of alcohol are major risk factors for certain cancers and significantly contribute to the rising incidence of inflammation-driven cancers such as hepatocellular carcinoma (HCC) and colorectal cancer (CRC) [5]. To date, most immunotherapies aim to enable cytotoxic T cells, members of the adaptive immune system, and reverse the dysfunction and/or exhaustion of these immune cells that frequently occur in the tumor microenvironment. More recently, innate immune cells, in particular natural killer (NK) cells, have emerged as promising candidates for immunotherapy [6]. NK cells can detect tumors without the need for antigen-specific receptors, as they recognize patterns specific to cancer cells. This allows them to recognize a broad range of changes present on malignant cells that are not present in the body’s normal cells and to respond rapidly.

Innate lymphoid cells (ILCs) are a family of cells that includes circulating NK cells and several subsets of tissue-resident ILCs that are positioned at barrier surfaces and within tissues, allowing them to rapidly recognize and respond to dangers such as infection, tissue damage or cancer. This tissue-resident feature of ILCs positions them within the parenchyma of tissues such as the liver, pancreas and intestine, where they are involved in immunosurveillance and can protect against disease. More recently, these populations have been found in significant numbers infiltrating cancer sites early in the development of the tumor, suggesting that this could be an important developmental checkpoint [7, 8]. This positioning provides a potential opportunity in immunotherapy to target non-T-cell populations that can facilitate elimination of tumor cells much earlier than might be possible for T cells. Thus, therapies could be engineered to be effective very early in a response to prevent tumor escape and metastasis.

ILCs within tumors have been shown to be highly heterogeneous. Although they can infiltrate tumors early in tumor establishment, their role(s) have been debated, as they have been shown to drive both protumor and antitumor responses [9]. While these divergent outcomes may depend on the type and stage of cancer, until recently, there has been little clarity around the overarching role of ILCs, whether they contribute positively or negatively to tumor development, and what determines the outcome in different settings. Understanding how ILCs shape the complex interplay between immune cells and tumor cells within the tumor microenvironment will help identify cellular interactions and signaling pathways that might be therapeutically targeted to enable immune cell self-protection and prevent tumor establishment.

## Innate lymphoid cells

Innate lymphoid cells (ILCs) are a heterogeneous immune cell population that was identified in the late 2000s by several teams [10–17]. Previously known lymphoid tissue inducer (LTi) cells and natural killer (NK) cells, discovered in 1997 and 1975, respectively, are also members of the ILC family [18, 19]. The diversity and heterogeneity of ILCs have only recently been described in detail. ILCs are enriched in mucosal tissues, where they fortify epithelial barrier defenses, or are positioned within tissues and are known as ‘tissue-resident’ cells. These lymphocytes lack adaptive antigen receptors, such as rearranged antigen-specific T- and B-cell receptors, but despite this, a number of their effector functions are similar to those of T cells [20]. In addition, they express receptors for cytokines and metabolites or peptides that allow them to detect danger signals and rapidly respond to changes in the microenvironment to orchestrate immune responses, including antitumor responses. ILCs also play an important role in rapidly responding to tissue damage by initiating tissue repair processes to maintain barrier integrity and homeostasis [21].

ILCs can be broadly divided into three main categories – group 1, 2 and 3 ILCs – which mirror the function of the CD4^+^ T helper (T_H_) cell subsets T_H_1, T_H_2 and T_H_17, respectively (Table 1). Group 1 ILCs include NK cells and ILC1s, which express the transcription factor T-BET encoded by *T-box transcription factor* 21 (*Tbx21*). Group 1 ILCs secrete cytokines such as interferon (IFN)-γ and tumor necrosis factor (TNF)-α that are critical in the immune response against viruses and tumors [22]. ILC2s are crucial in protection against helminths and parasites [23]. They produce interleukin (IL)-4, IL-5 and IL-13 and express the transcription factors GATA-binding protein 3 (GATA3) and RAR-related orphan receptor (ROR)-α [23]. Group 3 ILCs include three subsets of cells, natural cytotoxicity receptor (NCR)^+^ and NCR^−^ ILC3s and LTi cells, which all depend on the transcription factor RORγt. ILC3s secrete interleukin (IL)-17 and IL-22 and protect against extracellular bacteria [24]. Collectively, ILC1s, ILC2s and ILC3s have been termed ‘helper ILCs’, reflecting their similarities to CD4^+^ T helper cells. In contrast, NK cells, which share several functional features with CD8^+^ cytotoxic T cells, have been termed ‘cytotoxic ILCs’ [25].

**Table 1:** Phenotypes and functions of innate lymphoid cells in humans and mice

	Group 1 ILCs		Group 2 ILCs	Group 3 ILCs		
Subsets	NK cells	ILC1s	ILC2s	NCR^+^ ILC3s	NCR^−^ ILC3s	LTi cells
Effector function/mediators	IFN-γ, granzymes, perforin		IL-4, IL-5, IL-13, IL-9, AREG	IL-17, IL-22, GM-CSF		RANK, lymphotoxin, TNF-α, IL-17, IL-22
Transcription factors	T-BET, EOMES	T-BET, EOMES^h^	GATA3, RORα	RORγt, AhR		
Surface markers						
Mouse	CD49b, NK1.1, NKp46, KLRG1, CD122	CD49a, NK1.1, NKp46, TRAIL, CD200R, CD122, *CD127*	CD127, KLRG1, CD25, ST2	CD127, NKp46,	CD127, CCR6, *CD4*	CD127, CCR6
Human	CD16, CD56, NKp46, NKp30, NKp80, KLRG1, KIR, CD122, *CD127*	NKp46, TRAIL, CD200R, CD122 *CD49a*, *CD127*	CD127, KLRG1, CD25, CD161	CD127, NKp46, CCR6, CD56, NKp44	CD127, CCR6	CD127, CCR6, CD7

Italics, molecules that are not consistently expressed or show variable expression or expression only on specific subsets

h, expression in human cells only

Group 1 ILCs are of particular interest in cancer biology due to their well-described role in cancer immunosurveillance [26]. In humans, group 1 ILCs are defined as CD56^+^CD3^−^ immune cells (NKp46^+^CD3^−^ lymphocytes in mice), and markers that differentiate NK cells from ILC1s are highly tissue- and species dependent [27, 28]. Historically, NK cells and ILC1s were grouped together, but more recently, it has been found that they exhibit distinct differences and develop from separate progenitors. Thus, it is now recognized that NK cells and ILC1s are distinct cell types, but how they are regulated is not yet fully understood. In the context of controlling cancer, NK cells have been shown to be important in regulating the antitumor response through the elimination of transformed cells. NK cells can recognize infected, stressed, or transformed cells through the expression of a number of activating and inhibitory receptors, leading to the expression of cytotoxic effector proteins that enable target cell lysis. NK cells can be found in peripheral organs but typically circulate around the body, where they scan for abnormal cells that express reduced levels of major histocompatibility complex (MHC) class I and high levels of ligands associated with tissue damage or infection [29]. ILC1s, in contrast to NK cells, are usually found only in tissues and are thus tissue-resident, reflecting their relatively limited mobility beyond the local tissue environment and positioning them for defense within the tissues themselves [30]. Bernink et al. (2013) first demonstrated that ILC1s were distinct from NK cells by identifying that human intestinal ILC1s lacked expression of the NK cell signature molecules perforin and granzyme B, as well as expression of the IL-15 receptor alpha subunit (IL-15Rα) [31], which is essential for NK cell development [32, 33]. Although NK cells and ILC1s rely on the expression of different transcription factors during development, a degree of plasticity has been shown in phenotype and function of NK cells and ILC1s. An intermediate population of group 1 ILCs was identified in a model of cancer, and these cells expressed markers of both NK cells and ILC1s. These cells have not yet been well described, but their presence has been associated with exacerbated tumor growth, suggesting that they exhibit protumor functions. It is unclear whether intermediate ILC1s are protumorigenic or whether the exacerbated disease is a consequence of a reduced number of functional NK cells with tumoricidal activity [34].

Although it has been shown that NK cells and ILC1s coexist within tissues and can differ in their functions, several questions about the factors that distinguish NK cells from ILC1s remain. Whether the distinction between the two cell types should be based on their developmental pathways, functions, or phenotypes remains a matter of debate. Further complicating this challenge, many functions historically attributed to NK cells may in fact be mediated by ILC1s, such as immune memory in contact hypersensitivity models [35], cytotoxicity against activated hepatic stellate cells [36] and elimination of activated CD4^+^ T cells in chronic viral infections [37]. Although some phenotypic markers are shared across tissues, there are organ-specific differences that indicate that group 1 ILCs have distinct functions depending on their location in the tissue [38].

## Tissue-specific distribution of ILCs

The exposure of tissues to many different physiological signals has a major impact on the phenotype and function of ILCs that reside within different tissue-specific niches, allowing them to rapidly respond to local environmental changes. In healthy individuals, these ILCs have protective functions and are crucial in maintaining homeostasis [39]. When dysregulated, ILCs can contribute to pathology and tissue damage, but the mechanisms leading to these outcomes are unclear.

### The liver – An organ enriched in group 1 ILC subsets

**Fig. 1:**
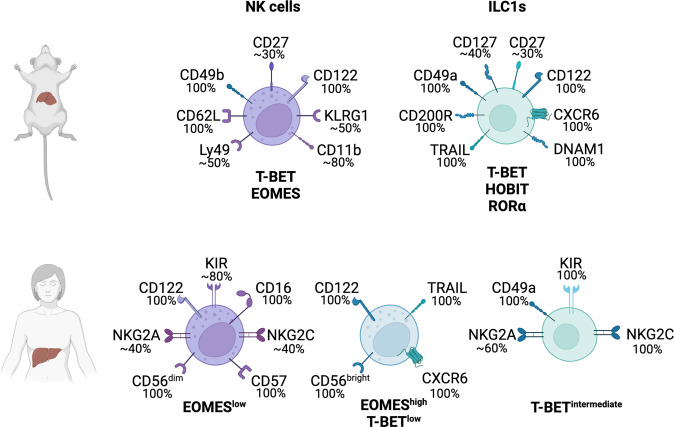
Receptor expression on mouse (top) and human (bottom) hepatic group 1 ILCs. Key transcription factors and the percentage of NK cells and ILC1s expressing each surface receptor

Liver-resident immune cells are constantly exposed to antigens carried in the blood through the portal vein to the liver. These include nutrients and antigens from the spleen and gastrointestinal tract. It would seem appropriate then, that the liver has a specialized framework of ILCs to mediate tissue protection. Understanding how these cells become dysregulated and the mechanisms by which this dysregulation that results in pathology occurs is important for the development of therapies designed to harness ILCs in this setting. Group 1 ILCs are found in multiple organs, but their phenotype and functions are highly tissue dependent. They are preferentially enriched in the liver, where they are thought to be part of the protective strategy for maintaining liver homeostasis. Group 1 ILCs comprise greater than 30% of liver-resident lymphocytes in humans (~6% in mice) compared with the quite small population (2%) detected in peripheral blood [27, 28]. Liver group 1 ILCs are composed of tissue-resident ILC1s and circulating NK cells. NK cells are identified by the expression of the integrin CD49b, also known as DX5, while ILC1s lack CD49b and instead express CD49a (Fig. 1) [35]. In addition to CD49a, hepatic ILC1s in mice express TNF-related apoptosis-inducing ligand (TRAIL) and the transmembrane glycoprotein CD200R (Fig. 1) [40, 41]. These receptors complement markers to identify ILC1s, as it has been shown that NK cells can upregulate CD49a in response to TGF-β [34, 42]. In addition, ILC1s can be distinguished from NK cells, as they lack CD62L and most inhibitory Ly49 receptors (Fig. 1) [33, 41]. Liver ILC1s were initially thought to be a subset of immature NK cells [43]. However, Peng et al. (2013) showed that they are indeed a separate subset with distinct functions rather than NK cell precursors, as had been previously proposed [35]. NK cells depend on the transcription factor nuclear factor, interleukin 3 regulated (NFIL3) and eomesodermin (EOMES), while ILC1s, previously termed liver-resident (lr) NK cells, develop normally in mice lacking these factors (Table 2) [44, 45]. Similar to other cells, ILC1s are strictly tissue-resident [30, 35] and express high levels of the *Cxcr6* transcript [46], which may contribute to their tissue residency through the interaction with CXCL16-expressing liver sinusoid cells [47]. Further in-depth characterization of liver group 1 ILCs revealed that ILC1s express similar levels of IFN-γ to NK cells following stimulation with PMA and ionomycin but higher levels of TNF-α and granulocyte-macrophage colony-stimulating factor (GM-CSF) [33]. Splenic and hepatic group 1 ILCs are similar in their phenotype and function but differ in their distribution. In the spleen, ILC1s are rare, comprising only approximately 1–2% of all group 1 ILCs, whereas approximately 30% of hepatic group 1 ILCs are ILC1s [40].

**Table 2:** Impact of loss of transcription factor expression on hepatic NK cells and ILC1s

Transcription factor	Mouse line	NK cells	ILC1s	Ref.
T-BET	*Tbx21*^*−/−*^	• Loss of mature cells • Higher cell turnover • Reduced cytotoxicity	• Almost complete loss	[68, 99]
EOMES	*Ncr1*^*Cre*^ *Eomes*^*fl/fl*^	• Reduced number • Loss of mature cells	• No impact	[45, 102]
	*Vav*^*Cre*^ *Eomes*^*fl/fl*^	• Reduced number	• No impact	[68]
HOBIT	*Hobit*^*−/−*^	• No impact	• Almost complete loss	[46, 64]
NFIL3	*Nfil3*^*−/−*^	• Almost complete loss	• No impact	[44]
AHR	*AhR*^*−/−*^	• No impact	• Significant reduction • Increased apoptosis	[110]
RORα	*Ncr1*^*Cre*^ *Rora*^*flfl*^	• No impact	• Significant reduction • Reduced expression of activating receptors • Reduced effector functions	[111]

The distinction between NK cells and ILC1s in the human liver is not as clearly defined as in the mouse. Human liver group 1 ILCs can be divided into three subsets depending on their EOMES expression: (1) EOMES^−^ cells that express CD49a and intermediate levels of T-BET are CD16^−^, CD57^−^, natural killer group 2 (NKG2)C^+^ and are capable of producing granzymes and cytokines [48]; (2) EOMES^low^ cells that phenotypically resemble peripheral blood NK cells with high levels of CD16 and cytotoxic molecules have intermediate expression of CD56 and lack CXCR6; and (3) EOMES^high^ T-BET^low^ cells that express TRAIL and CXCR6 produce lower levels of granzyme B and perforin and appear to be tissue-resident (Fig. 1) [49, 50]. Phenotypically, the EOMES^−^ and EOMES^high^ T-BET^low^ subsets resemble mouse ILC1s, while EOMES^low^ cells exhibit features of NK cells, most notably high expression of cytotoxic molecules. The EOMES^high^CXCR6^+^ subset has been shown to be present in the fetal liver, as well as other fetal organs, such as the lung and spleen, suggesting that these cells arise prenatally and are not restricted to the liver but are a tissue-resident subset present in multiple organs before birth [51]. Distinct differences exist between human and mouse ILC1s. First, a subset of human hepatic ILC1s express high levels of EOMES, whereas mouse ILC1s are defined by the lack of EOMES expression (Fig. 1) and develop in EOMES-deficient mice [45]. EOMES^+^ ILC1s have been described in other organs, such as the human intestine, where ILC1s express high levels of *EOMES* transcript [52], suggesting that there are species-specific differences in the regulation and function of EOMES. Second, CD49a is used as a marker to define hepatic ILC1s in the liver, but its expression is limited to a small subset of group 1 ILCs in the human liver (Fig. 1) [48]. Transcriptomic analyses of mouse and human hepatic CD49a^+^ group 1 ILCs showed that some genes were differentially expressed: human CD49a^+^ cells expressed higher levels of *GZMK* and *PRF1*, while their mouse counterparts had higher expression of *Ifng*, *Gzma* and *Gzmb* [53]. The production of cytotoxic mediators by both groups of cells suggests that they have overlapping functions. CD49a^+^EOMES^+^ ILC1s have been described in mucosal tissues and tumors [52, 54], but it is unclear whether the CD49a^+^EOMES^−^ subsets are present in any other tissues or restricted to the hepatic environment.

### The mucosal environment and dampened group 1 ILC cytotoxicity

In mucosal tissues, such as the lung and intestine, NK cells are functionally distinct from those in the spleen and liver and characterized by lower production of cytokines and cytotoxic molecules [55, 56]. Mature lung NK cells, defined as CD56^dim^ CD16^+^ cells in humans and CD27^−^CD11b^+^ cells in mice, show little cytotoxicity against target cells and reduced expression of the degranulation marker CD107a [55]. In the mouse lung, NK cells express higher levels of inhibitory receptors and lower levels of activating receptors than splenic NK cells [28]. This hyporesponsive state of NK cells in naïve lungs is most likely critical to maintaining homeostasis and preventing overresponsiveness to harmless antigens in the airways. The mechanisms leading to this hyporesponsiveness are unclear, but it is likely that the cytokine *milieu* largely shapes local differences in NK cell function. Mouse intestinal NK cells produce IFN-γ, granzyme B and perforin, albeit at lower levels than splenic NK cells, despite their similar close positioning to a mucosal barrier surface to pulmonary NK cells [56]. Intestinal NK cells can respond quickly to bacterial stimuli to induce an IFN-γ response and provide protection against infections such as *Citrobacter rodentium* and *Salmonella enterica* infections [57–59]. In addition to NK cells, a subtype of ILC1 distinct from NK cells have been identified in the intestinal mucosa. These ILC1s are IL-12- and IL-15-responsive, produce IFN-γ [52] and are enriched in the mucosa of Crohn’s disease patients, where they are thought to contribute to pathological inflammation [31, 52]. The integrin profile of these ILC1s resembles that of tissue-resident memory (T_RM_) CD8^+^ T cells, which also express CD103 and β7 integrin, together forming the αEβ7 heterodimer that binds to E-cadherin on epithelial cells and the collagen-binding integrin CD49a. This similarity suggests that they are strictly tissue-resident and are retained in the intestinal epithelium [52]. Pulmonary ILC1s express the tissue residency marker CD69 in the human lung and have variable expression of CD103 and CD49a, possibly indicating different stages of tissue residency. In contrast to IFN-γ-producing intestinal ILC1s, pulmonary ILC1s produce CCL5, macrophage inflammatory protein (MIP)-1β and GM-CSF following stimulation with IL-15, perforin and granzyme B, underlining the distinct functional differences between ILC1s in different tissue locations [60].

### NK cells and ILC1s – different cell states or cell types?

Although NK cells and ILC1s are now recognized as the two different ILC subsets, how their roles differ in the body remains unclear. Initially, it was thought that they differed in their cytotoxicity and tissue residency characteristics, with ILC1s believed to be noncytotoxic and tissue resident and, in contrast, NK cells believed to be cytotoxic and circulating. It is now recognized that a number of key molecules distinguish NK cells from ILC1s. It should be noted, however, that expression of these markers can be highly tissue- and context dependent. For example, CD49a, an integrin previously used to identify hepatic and splenic ILC1s, can be upregulated on NK cells in response to TGF-β [34, 42]. It is also now clear that ILC1s can be cytotoxic and that they can enter the circulation in different settings [61–66]. In addition to the mapping of surface molecules, NK cells and ILC1s can be distinguished by the expression of transcription factors. In mice, NK cells typically express both T-BET and EOMES, whereas ILC1s express T-BET and lack EOMES. Forced expression of EOMES under the *Tbx21* (encoding T-BET) promoter completely abolishes CD49a^+^CD49b^−^ ILC1s, leaving only CD49a^−^CD49b^+^ NK cells. This suggests that EOMES expression is sufficient to induce an NK cell phenotype in hepatic ILC1s and that it can indeed be used as a marker for NK cells [45]. This is in contrast to human ILC1s, which express EOMES. The function of EOMES in these cells is unclear, and whether EOMES may have species-specific functions is a matter of ongoing research. EOMES expression in tissue-resident group 1 ILCs that are otherwise phenotypically and functionally similar to ILC1s suggests that it may not be a definitive marker to distinguish NK cells from ILC1s. In fact, in-depth analysis of hepatic ILC1s and NK cells revealed that the mouse liver contains a population of CD49a^+^ ILC1s that expresses low levels of EOMES [67].

Contributing to the lack of clarity around ILC1 and NK cell identity is their ability to adopt flexible functional programs within the tissues. Adoptive transfer of purified hepatic TRAIL^+^ ILC1s or CD49b^+^ NK cells into immune-deficient *Rag2*^*−/−*^*Il2rg*^*−/−*^ mice that lack both adaptive and innate lymphocytes revealed that TRAIL^+^CD49b^−^ cells can give rise to EOMES^+^ cells, while CD49b^+^TRAIL^−^ cells were unable to develop into TRAIL-expressing cells [68, 69]. In contrast, recent in vivo studies have shown that ILC1s do not give rise to NK cells in the adult liver. Hepatic NK cells had no history of HOBIT expression, a transcription factor expressed by all hepatic ILC1s [64]. NK cells can adopt an ILC1 phenotype in cancer, infection and obesity [34, 42, 70], but does this mean they are truly ILC1s or alternately NK cells expressing markers that were previously attributed to ILC1s? Currently, most studies rely on a combination of the expression of surface markers and transcription factors and cell function to identify ILC1s and NK cells. Tools such as single-cell sequencing, fate mapping and transfer of progenitor cells will provide greater clarity around group 1 ILC identity.

## ILC2s and ILC3s – rare but ready to react

While group 1 ILCs are by far the most abundant type of ILC in the liver, small numbers of hepatic ILC2s also exist. In the intestine, ILC2s reside within the lamina propria and accumulate specifically in the villi and lymphatic vessels within the villi following inflammation, allowing rapid redistribution to the periphery via the lymphatic system [71]. In the lung, ILC2s are situated in close proximity to blood vessels and airways but are absent from alveoli [72, 73]. This positioning allows them to react to environmental cues such as antigens in the airways and limits exposure of alveoli to potentially tissue-damaging inflammatory stimuli. Following inflammatory cues, such as allergic inflammation, ILC2s traffic to the parenchyma, where they mediate effector functions [74]. Similarly, hepatic ILC2s are located near the vasculature [73], suggesting that they can not only sense local cues but also respond to systemic changes and signals in the bloodstream. The phenotype of ILC2s across tissues varies, and sequencing analyses have revealed differences in the expression of cytokine receptors depending on their specific tissue location. Lung and liver ILC2s have been shown to express IL-33R, while intestinal ILC2s fail to express IL-33R but express IL-25R, indicating differences in responsiveness depending on the tissue location [75]. In helminth infections, ILC2s play a key role in the clearance of worms from the gut and lung. Intestinal and pulmonary tuft cells are triggered by helminths to secrete IL-25 and cysteinyl leukotrienes, which in turn activate ILC2s to initiate a protective type 2 immune response [76, 77]. In addition to this acute immune response, ILC2s play a key and initially unexpected role in tissue repair following helminth or viral infections. This is mediated by upregulation of growth factors such as amphiregulin and the anti-apoptotic protein B-cell lymphoma 3-encoded protein (BCL-3) [78, 79]. In contrast to these protective functions, activation of ILC2s can result in pathology such as IL-13-mediated airway hyperreactivity during viral infections [80] or allergic asthma [81, 82] due to overproduction of type 2 cytokines. Liver injury can lead to IL-33 production by stressed hepatocytes [83], and mouse ILC2s rapidly accumulated and acquired an activated phenotype in response to treatment with IL-33. The ILC2s expressed high levels of killer cell lectin-like receptor G1 (KLRG1) and GATA3 and produced type 2 cytokines, such as IL-4, IL-5 and IL-13. The accumulation of ILC2s was inhibited by IFN-γ, suggesting that ILC2s only expand in response to a very specific local cytokine *milieu* [84]. In human patients, the frequency of ILC2s was increased in fibrotic or cirrhotic livers, which was associated with increased levels of plasma IL-33 [85].

Similar to ILC2s, ILC3s are rare in the healthy liver but can expand in the disease setting. In hepatitis B virus (HBV)-related chronic liver disease and a mouse model of fibrosis, the frequency of IL-17A- and IL-22-producing ILC3s was found to be increased up to twofold [86]. Using a mouse model of HCC, Liu et al. (2019) found that NCR^−^IL-17-producing ILC3s accumulated in the presence of IL-23, promoting disease progression. In the same model, adoptively transferred ILC1s acquired RORγt expression in the tumor microenvironment, suggesting that the increase in ILC3s might be a result not only of expansion of the ILC3 population residing in the naïve liver but also of plasticity allowing the transition of ILC1s into ILC3s [87]. In contrast to the liver, other tissue environments, in particular mucosal tissues, favor the accumulation of ILC3s. ILC3s are the most abundant subset of ILCs in the intestine and play a critical role in the maintenance of gut homeostasis. They are found in the intestinal lamina propria and villi but differ from ILC2s in that they form clusters at the base of intestinal villi near Peyer’s patches and are known as cryptopatches [88–90]. Their proximity to other immune cells in Peyer’s patches and cryptopatches may reflect their function as antigen-presenting cells [91]. ILC3s, in contrast to ILC2s, are highly dependent on the composition of the microbiome along the intestinal tract [75]. The microbiota is pivotal for the induction of IL-22 production required for mucosal immune protection [14, 92]. A lack of IL-22-producing ILC3s results in bacterial dissemination and systemic inflammation [93]. In inflammatory conditions, such as inflammatory bowel diseases, ILC3s can become dysregulated, resulting in the production of excessive levels of IL-17A and IL-22 that contribute to disease pathology [94–96]. Pulmonary ILC3s are less well studied than intestinal ILC3s due to their reduced prevalence in mouse lungs, where they comprise only ~2% of all ILCs [97]. In the human lung, ILC3s make up more than half of all ILCs [98], highlighting potential species-dependent differences in tissue environments.

## Transcriptional regulation of hepatic NK cells and ILC1s

The regulation of group 1 ILCs is complex, and several transcription factors dictate the phenotype and function of NK cells and ILC1s. Experiments in mice that are deficient for one or more transcription factors have increased the understanding of the transcriptional regulation of hepatic ILCs. Germline deletion of T-BET, encoded by *Tbx21*, results in a striking, but incomplete, loss of group 1 ILCs in both the spleen and liver. Analysis of the residual NK cells showed a complete loss of TRAIL^+^ ILC1s and CD11b^+^CD27^+^ mature NK cells, suggesting that T-BET is essential for ILC1 development and NK cell maturation (Table 2) [68]. The loss of *Tbx21* was associated with higher turnover of NK cells and reduced cytotoxicity against target cells, further corroborating the finding that T-BET is important in mediating the maturation and effector functions of NK cells [99]. EOMES is essential for NK cell development and maturation but is not required for ILC1s in mice (Table 2). *Ncr1*, encoding NKp46, is expressed by NK cells, ILC1s and a subset of ILC3s (NCR^+^ ILC3s). The *Ncr1*^*Cre*^ mouse has proven invaluable for studying the function of transcription factors and other proteins in these subsets [100, 101]. *Ncr1*^*Cre*^*Eomes*^*fl/fl*^ mice, which lack EOMES only in *Ncr1*-expressing cells, showed significant defects in the NK cell compartment with a marked reduction in CD49a^−^CD49b^+^ cells in multiple tissues, including the liver, spleen, salivary gland and lung (Table 2). In contrast, the CD49a^+^CD49b^−^ ILC1 population remained unaffected. Thus, ILC1s appear to develop and be maintained independent of EOMES, while NK cells rely on EOMES expression [45]. Despite a marked reduction in CD49b^+^ cells in *Ncr1*^*Cre*^*Eomes*^*fl/fl*^ mice, a small number of cells remained. In a separate study, NK cells in the spleen and bone marrow of *Ncr1*^*Cre*^*Eomes*^*fl/fl*^ mice were further characterized and shown to be mostly immature, suggesting an additional function for EOMES in NK cell maturation [102]. In a second mouse model in which EOMES is deleted in all hematopoietic cells, including common lymphoid progenitors (CLPs) (namely *Vav*^*Cre*^*Eomes*^*fl/fl*^ mice) [103], lineage^−^NK1.1^+^NKp46^+^CD122^+^ cells were reduced in the liver and spleen [68]. The specific reduction in CD49b^+^TRAIL^−^ cells in the liver suggests that NK cells were lost, while ILC1s were retained (Table 2). Nonetheless, a population of cells expressing low levels of CD49b also remained in the liver of *Vav*^*Cre*^ *Eomes*^*fl/fl*^ mice, similar to that in *Ncr1*^*Cre*^*Eomes*^*fl/fl*^ mice [68]. Is it unclear whether these cells truly develop independent of EOMES or whether their presence is due to incomplete Cre-mediated deletion of *Eomes*.

**Fig. 2:**
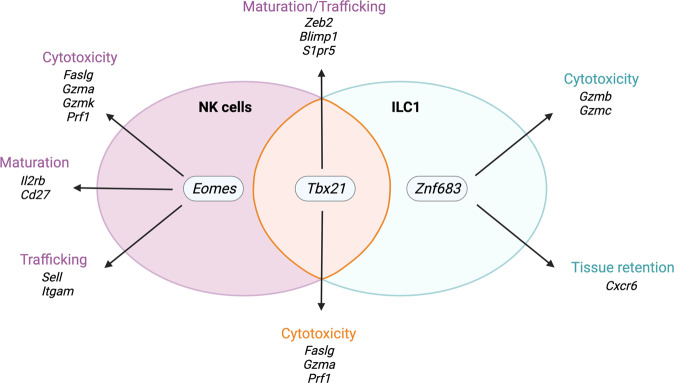
Expression of transcription factors and their target genes in hepatic NK cells and ILC1s in mice. *Eomes* expression is unique to NK cells (purple) and induces the expression of genes that are involved in cytotoxicity, maturation and trafficking. *Tbx21* is expressed by both cells (orange) and induces the expression of genes associated with cytotoxicity in both cell types and genes associated with maturation (*Zeb2, Blimp1*) and trafficking (*S1pr5*) in NK cells only, possibly due to the different expression levels or coregulation by other transcription factors. *Znf683* is only expressed by ILC1s (teal) and regulates cytotoxicity and tissue retention

How do EOMES and T-BET regulate the development of group 1 ILC1s? CD122, which is a subunit of the IL-15 receptor, is expressed on the surface of NK cells and their precursors, where it regulates maturation and survival in response to IL-15 [102]. In T cells, EOMES binds directly to the *Il2rb* promoter and induces CD122 expression (Fig. 2) [104]. Similarly, EOMES-deficient NK cells express reduced levels of CD122, resulting in developmental and functional defects [102]. While NK cells strictly rely on IL-15 signaling, ILC1s are retained in some organs of *Il15ra*^*−/−*^ mice. ILC1s are absent in the liver but not the intestine of *Il15ra*^*−/−*^ mice, suggesting that the requirements for IL-15 are highly tissue dependent [33, 52]. Similar to EOMES, T-BET can regulate CD122 expression on T cells [99], but this has not been studied in the context of group 1 ILC biology. How IL-15 responsiveness is regulated in ILC1s that do not express EOMES is thus unclear.

In addition to CD122, EOMES was shown to induce the expression of genes involved in trafficking, such as *Itgam* (encoding CD11b) and *Sell* (encoding L-selectin CD62L), and cytotoxic mediators, such as Fas ligand (FasL) and granzyme K (Fig. 2). It also repressed *Zfp683*, which encodes HOBIT and is crucial for liver ILC1 development [46, 102]. T-BET induced the expression of some other known repressors, such as *zinc finger E-box binding homeobox 2* (*Zeb2*) and *B-lymphocyte-induced maturation protein 1* (*Blimp1*), which are important for NK cell maturation (Fig. 2) [105, 106]. T-BET was found to repress *transcription factor 7 (Tcf7*, which encodes TCF-1) and granzyme B (*Gzmb)* expression [107]. Thus, repression of TCF1 is crucial for normal NK cell processes, which include the production of granzyme B. T-BET was further shown to bind to the *sphingosine-1-phosphate receptor 5* (*S1pr5*) locus to induce the expression of S1P5, which is critical for egress from the bone marrow [108]. Despite their distinct functions, the DNA binding domains of EOMES and T-BET are highly similar, suggesting that they bind to the consensus sequence and share some functions. *ll7r* was found to be corepressed by both EOMES and T-BET, resulting in higher levels of expression of IL-7 receptor (CD127) in EOMES- and T-BET-deficient NK cells. T-BET^+^ ILC1s express CD127 in some organs, such as the intestine, suggesting that T-BET alone is not sufficient to suppress *Il7r* expression, while NK cells, which express both EOMES and T-BET, are CD127^−^. Coinduced genes included those associated with cytotoxicity, such as *Gzma*, *Faslg* and *Prf1*, which were downregulated in both T-BET- and EOMES-deficient mice (Fig. 2). Despite the similarities in their binding sequences, T-BET and EOMES can differentially regulate certain genes, including *Cd27* and *Cd69*. EOMES-deficient NK cells expressed lower levels of *Cd27* and *Cd69* at the protein and transcription levels, while their expression was increased in the absence of T-BET. Both *Cd27* and *CD69* were highly expressed in immature NK cells but downregulated in mature cells, suggesting sequential activation of EOMES followed by T-BET in NK cell development [102]. How this sequential activation is regulated is not fully understood, but overexpression of T-BET in progenitor cells restricted the development of EOMES^+^ NK cells, suggesting that T-BET actively represses EOMES expression [109]. Similarly, in the absence of Notch signaling, EOMES expression was increased in liver ILC1s and was associated with higher production of IFN-γ and TNF-α and increased cytotoxicity. This finding suggests that EOMES might normally be actively repressed in ILC1s and thus, the phenotype of ILC1s is dependent on changes in the level of EOMES expression [67].

In addition to the reliance of ILC1s on T-BET, liver ILC1s strictly depend on the transcription factor zinc finger protein 683 (*Znf683*), also known as HOBIT (Table 2) [46]. HOBIT drives the differentiation of cKit^+^TCF1^+^ early ILC1s into granzyme B-expressing ILC1s and is therefore crucial for the maturation and function of hepatic ILC1s [64]. Lack of HOBIT resulted in the arrest of ILC1s in an immature state marked by high expression of *Il7r, Il18r1* and *Rora*, similar to the phenotype of ILC1s found in other tissues, such as the small intestine and mesenteric lymph nodes. ILC1s across different tissues expressed HOBIT, but HOBIT deficiency only led to a marked reduction in ILC1s of the liver and salivary gland. The authors argued that only the liver and salivary gland harbor granzyme B-expressing ILC1s and that ILC1s residing in other tissues are halted in a less mature state, suggesting that the local cytokine environment dictates the maturation stage of ILC1s. Lastly, the authors showed that NK cells had no history of HOBIT expression, suggesting that HOBIT is upregulated after commitment to the ILC1 lineage [64]. The aryl hydrocarbon receptor (AHR) is also involved in hepatic ILC1 development [110]. AHR is expressed at higher levels in hepatic ILC1s than in liver NK cells or NK cells in other organs. In one study, *Ahr*^*−/−*^ mice showed a significant decrease in CD49a^+^CD49b^−^ hepatic ILC1s (Table 2) [110]. Administration of AHR ligand resulted in increased ILC1s and reduced the expression of the inhibitory receptors T cell immunoglobulin domain and mucin domain 3 (TIM-3) and KLRG1 on group 1 ILCs in the liver. The frequency of apoptotic cells was reduced in mice that received AHR ligands compared to control mice, suggesting that AHR is required for the maintenance of liver ILC1s because it reduces the expression of inhibitory receptors and promotes survival [110]. A recent study identified RAR-related orphan receptor alpha (RORα) as a critical transcription factor involved in ILC1 development in the liver. *Rora* transcript levels were high in liver ILC1s but were not found in NK cells. Deletion of RORα in *Ncr1*- (all group 1 ILCs) or *Vav*-expressing cells (all hematopoietic cells) led to a defect specifically in CD49a^+^ ILC1s and reduced expression of activating receptors, reduced IFN-γ production and increased apoptosis, suggesting that the remaining ILC1s were highly dysfunctional (Table 2) [111].

## What does epigenetic regulation tell us about ILC function and plasticity?

In addition to transcriptional regulation, the epigenetic landscape of a cell dictates its function and phenotype. The chromatin structure and epigenetic modifications of ILCs reflect their function and can indicate whether a cell exists in a plastic state and retains the capability of mediating different effector functions or whether it is terminally differentiated. Certain ILC subsets have been described to exhibit a degree of plasticity, which means that one subset can acquire the phenotype and function of a different subset. Examples of this include the NK-cell-to-ILC1 plasticity that has been observed in models of cancer, liver disease and infection with *Toxoplasma gondii* [34, 42, 112, 113]. Understanding how the epigenetic landscape and chromatin structure change during ILC activation, plasticity, and dysfunction and whether these changes can be reversed is of interest for the development of new therapies targeting ILCs.

Early studies showed that certain loci in ILCs are constitutively accessible, even in the absence of stimulation or cytokine production [114], indicating that ILCs are preprimed to respond quickly to an insult or stimulus without the need for extensive reconfiguration of their molecular machinery. In fact, the chromatin structure of ILCs is more comparable to that of activated T cells than to that of naïve cells, suggesting that the function of ILCs is aligned with their capacity for rapid responses [115]. By comparing the chromatin and transcriptional landscapes of different ILC subsets in various tissues, Shih et al. (2016) demonstrated that the lineage-specific chromatin landscape was closely correlated with the functionality of different subsets. For example, the *Ifng* locus was accessible in NK cells and ILC1s isolated from the spleen and liver, whereas the *Il13, Il5* and *Il9* loci were accessible in intestinal ILC2s, and the *Il17a, Il17f*, and *Il22* loci were accessible in intestinal ILC3s. However, some regulatory elements were found to be accessible even if the transcript was not expressed. *Eomes* and *Gata3* were accessible in all cell types, *Tbx21* was accessible in NCR^+^ ILC3s, and *Rorc*, encoding RORγt, was accessible in ILC2s and partially accessible in ILC1s, suggesting that these genes can be easily activated [115]. On a broader level, the authors found that 25% of accessible regulatory elements were shared by all subsets, and 75% were unique to an individual subset or alternately shared by several subsets [115]. This finding suggests that while the chromatin structure and gene accessibility reflect the function of different ILC subsets, some genes that are not expressed remain accessible, and this could explain the ability of some subsets to switch to effector functions normally associated with other ILC subsets.

A study specifically comparing the accessibility of genes in NK cells and ILC1s isolated from the liver, spleen and salivary gland showed that among the differentially regulated genes, *Sell, S1pr1* and *S1pr5* were more accessible in NK cells, while *Itga1, Gzmc* and *Cxcr6* were accessible in ILC1s. This result reflects the expression pattern of these genes and shows that genes regulating the trafficking and effector functions of group 1 ILCs are not readily accessible across different subsets [66].

## How does the cytokine *milieu* impact the epigenetic landscape of ILCs?

Pathologies, including cancer, are often associated with drastic changes in the cytokine *milieu*. It is well established that this can modulate the effector function of ILCs, but how cytokines impact the chromatin structure is less well understood. Comparing chromatin accessibility before and after stimulation revealed that fewer ATAC-seq sites were available after stimulation, suggesting that ILCs exist in a state in which they can easily acquire different effector functions and become more restricted in their functions following stimulation [115]. Other factors, such as environmental cues or the location within the tissue, may influence the accessibility of some genes to impact the effector functions of ILCs. In addition, antibiotic treatment did not change the global chromatin structure but dramatically affected the methylation status of subset-specific enhancers [116]. Shih et al. (2016) undertook a combined analysis of several datasets including ATAC-seq data from group 1 ILCs isolated from different tissues, including spleen, liver and bone marrow. The researchers showed that regardless of the different tissue origins of cells, ILCs clustered together, suggesting that the tissue microenvironment has little impact on the chromatin structure [115]. This could be explained by the fact that chromatin landscapes are established early during ILC development, possibly before exit from the bone marrow [115]. Collectively, these studies highlight that even though each ILC subset has its own subset-specific epigenetic signature and chromatin structure, nonsignature genes are accessible, allowing ILCs to quickly adapt to changing tissue environments. Studying how the epigenetic landscape of ILCs is impacted in the context of pathologies could help us gain a better understanding of the molecular mechanisms underlying ILC dysfunction and plasticity.

## Origins of liver-resident ILC1s and NK cells

Inflammation and tissue damage are often associated with an accumulation of immune cells within the tissue. This increase can be a result of local proliferation or recruitment of cells from the periphery. Most ILCs, apart from NK cells, do not typically circulate throughout the body but can be found in the peripheral blood of human patients in some contexts, such as cancer [62], psoriasis [117] and allergic asthma [118]. Circulating ILC2s were identified in a mouse model of helminth infection [71], but whether they are present in other contexts is unclear. This suggests that certain stimuli can induce de novo hematopoiesis in the bone marrow that results in an increase in ILCs that are recruited to the site of inflammation. Understanding how ILCs develop, at what stage during development they diverge and what cues induce hematopoiesis can highlight potential therapeutic targets.

The earliest ILC progenitor is α-lymphoid progenitors (αLPs) which are characterized by expression of CXCR6, CD127 and α_4_β_7_ [119, 120]. αLPs develop into programmed cell death protein 1 (PD-1)- and promyelocytic leukemia zinc finger protein (PLZF)-expressing ILC-committed progenitors (ILCps) that can give rise to all ILC subsets, including NK cells [121]. An ILC2-committed progenitor (ILC2p) has been described and can be identified by the expression of CD25 (*Il2ra*) [120, 122]. NK cell progenitors (NKPs) are characterized by the expression of CD122 (*Il2rb*), a component of the IL-2/IL-15-receptor complex, but it is unclear whether this population is the first committed progenitor and which population these cells are derived from. Whether a similar fate-committed subset exists for ILC1s and ILC3s and at what stage during development they diverge are subjects of ongoing research. It is unclear what stimuli drive commitment to certain ILC lineages. Understanding what induces a progenitor cell to develop into an NK cell as opposed to an ILC1 may reveal novel therapeutic targets that enable targeting of specific ILC subsets at early stages of development.

Although classic white blood cell differentiation occurs in the bone marrow, tissue-resident ILC progenitors have been identified in a number of other organs. This tissue-specific source of ILCs may be important in disease settings in which ILCs need to be recruited or replenished. The advantage of a tissue-resident progenitor over a progenitor residing in the bone marrow is that they can sense local cues and proliferate in the absence of a systemic response. Recent studies have identified Lin^−^Sca-1^+^Mac-1^+^ (LSM) cells as liver-resident progenitors in adults. These cells preferentially give rise to ILC1s over NK cells via an intermediate CD49a^+^CD122^+^ stage in an IFN-γ-dependent manner [123]. LSM cells exist in the fetal and adult liver but not the bone marrow, suggesting a liver-specific niche that allows the maintenance of an ILC1-specific precursor. Similarly, the adult mouse lung contains a subset of cells characterized by the expression of RORα, CD127 and IL-18Rα with differentiation potential similar to that of bone marrow ILCps [124]. These cells proliferate upon injury, suggesting that they can act as a local source of ILCs in settings of inflammation or tissue damage. Whether either of these progenitor cell types proliferates in the context of cancer is unknown.

In addition to those in the bone marrow and selected peripheral organs, circulating ILC progenitors have been described in humans and mice. A subset of CD117^+^ ILCs that lacks expression of transcription factors characterizing mature ILC subsets was defined in peripheral blood of healthy human individuals. These cells were able to give rise to all ILC subsets when cultured in vitro or transferred to humanized mice, suggesting that they are multipotent progenitors that can develop into functional ILCs [125]. Similarly, a population of ILC progenitors in the mouse lung was shown to be derived from the circulation, suggesting that ILC progenitors are, at least transiently, present in the peripheral blood of mice [126].

A recent study showed that hepatic ILC1s derived from different origins differ in their function [65]. By using inducible reporter mice to fate-map hematopoietic stem cells, but not lineage-restricted progenitors, the authors showed that a population of Ly49E^+^ ILC1s was primarily fetus-derived and decreased with age, while Ly49E^−^ ILC1s retained the ability to be replenished from adult progenitors. Functionally, Ly49E^+^ ILC1s expressed higher levels of granzymes and lysed target cells more efficiently in vitro, suggesting a distinct role for these fetus-derived cells in the host defense of neonates, whose adaptive immune system is not fully developed [65].

## ILCs in cancer

### NK cells

NK cells typically contribute to the antitumor immune response but have recently been implicated in driving disease progression in certain contexts. The infiltration of NK cells into solid tumors is often used as a prognostic marker of survival due to their ability to recognize and eliminate tumor cells without prior sensitization [127]. Various cancer immunotherapy approaches are based on NK cells or their mechanisms, including adoptive transfer of NK cells, chimeric antigen receptor (CAR) NK cells, T cells bearing NK cell receptors or antibodies resulting in NK cell activation [29]. However, the presence of tumor-infiltrating NK cells is not always beneficial. Over time, tumor cells can downregulate ligands that bind to activating receptors on NK cells, rendering them invisible to NK cells and thereby enabling them to evade NK-cell-mediated antitumor immunity [128]. Moreover, the tumor microenvironment can induce changes in NK cell function by modulating NK cells to express lower levels of activating receptors, such as NKG2D, and higher levels of inhibitory receptors, such as NKG2A [129, 130]. These changes are often correlated with poor prognosis, but whether this is solely a consequence of NK cell dysfunction or whether NK cells actively contribute to tumor progression is unclear.

### Antitumor function of ILC1s

Depending on the context and specific tumor type, ILC1s have been associated with both disease progression and paradoxically protective antitumor responses. In a mouse model of mammary tumors, tumor-infiltrating ILC1s producing high levels of granzyme B lysed tumor cells directly [61]. This was unexpected, as ILC1s were thought to be less cytotoxic than NK cells owing to their low expression of cytotoxic molecules and high expression of cytokines at steady state [109]. To confirm that this effect was mediated by ILC1s and not NK cells, the authors induced tumors in *Nfil3*^*−/−*^ mice, which have reduced numbers of NK cells but normal numbers of ILC1s within the mammary tissue, and showed that the reduction in NK cells did not result in an impaired antitumor response. Thus, ILC1s rather than NK cells appear to be the key cells driving the inhibition of mammary tumor growth through cytotoxic mechanisms. Strikingly, these ILC1s did not express CD127, a marker often used to identify ILCs [61], prompting the hypothesis that ILC1s can be grouped into ‘cytotoxic’ CD127^−^ ILC1s and ‘helper’ CD127^+^ ILC1s. In patients with colorectal cancer, a subset of CD127^−^ ILCs expressing high levels of cytotoxic molecules was identified, suggesting that cytotoxic ILC1s exist across species and in different tissues [131]. The authors identified three subsets of group 1 ILCs: a CD127^+^ ILC subset and two subsets expressing CD56 and lacking CD127. The latter could be distinguished by the expression of the different isoforms CD45RA and CD45RO. CD45RA^+^ CD45RO^−^ expression was thought to identify NK cells, whereas CD45RA^−^CD45RO^+^ cells clustered between NK cells and CD127^+^ ILCs. In that study, the authors showed that CD45RO^+^ cells expressed higher levels of granzyme B and perforin, suggesting that CD45RO^+^ cells are cytotoxic and contribute to the antitumor response, similar to the cells identified in mice by Dadi et al. (2016) [61, 131]. A recent study identified CD127^+^ ILC1s as noncytotoxic ILC1s that require expression of the transcription factor HOBIT to develop into CD127^−^ ILC1s with cytotoxic effector function [64]. The presence of CD127^−^ ILC1s within the tumor microenvironment suggests that local cues support the development or maintenance of these cells. Understanding what these cues are and how they can be modulated may help in the development of new therapies that aim to expand these antitumor cytotoxic ILC1s specifically. A subset of CD127^+^ ILC1s with cytotoxic activity was identified in acute myeloid leukemia (AML) patients. The elimination of tumor cells was independent of inhibitory killer Ig-like receptors (KIRs) but depended on NKp30 and NKp80, the blockade of which resulted in decreased cytotoxicity against target cells. The high expression of granzymes seen in these cells, in particular granzyme A, suggests that engagement of NKp30 and NKp80 triggers release of granzymes and subsequent target cell death. Similarly, blocking TRAIL reduced the specific lysis of target cells. Thus, these cells may contribute to antitumor immunity by inducing TRAIL- and granzyme-mediated target cell death. At diagnosis, these cells exhibited impaired function, but as patients entered remission, this phenotype was reversed, suggesting that these cells can indeed contribute to the anticancer immune response. The lack of cytotoxicity can be recapitulated in vitro in the presence of TGF-β and AHR ligands, which inhibits cytotoxicity [132]. ILC1s can further contribute to the control of AML by eliminating leukemic stem cells (LSCs) and inhibiting their differentiation into myeloid blasts [133]. These effects were dependent on the production of IFN-γ by ILC1s and cell-to-cell interactions between ILC1s and LSCs, suggesting that receptor–ligand interactions induce cytokine production by ILC1s. Indeed, blocking DNAM-1 and CD127 in a coculture of LSCs and ILC1s reduced the percentage of IFN-γ-producing ILC1s, suggesting that LSCs express ligands for these receptors. Functional impairment of ILC1s in AML patients may lead to outgrowth of LSCs and disease relapse [133]. In chromophobe renal cell carcinoma (chRCC), cytotoxicity against tumor cells mediated by ILC1s was shown to be dependent on the production of IL-15 by the tumor cells [134]. Thus, targeting ILC1s may be a promising therapeutic approach in a number of cancers.

### Protumor function of ILC1s

While ILC1s can act to restrict or eliminate tumors, they have also been found to promote tumor development in some settings. In human non-small-cell lung carcinoma (NSCLC), disease progression was associated with an increase in a subset of group 1 ILCs that express low levels of EOMES. This was attributed to an increase in non-NK ILC1s with reduced antitumor activity [135]. High expression of ILC1-related genes was associated with decreased survival in clear cell renal cell carcinoma (ccRCC) patients [134]. Whether ILC1s actively contribute to cancer progression or alternately their accumulation is a consequence of immune cell remodeling within the tumor microenvironment was not addressed in these studies. In AML, the frequency of ILC1s was increased, while NCR^+^ ILC3s were reduced in the peripheral blood of patients compared with healthy controls. In addition, IFN-γ and TNF-α production by ILC1s within the tumor was impaired, suggesting that the tumor-infiltrating ILC1s may be dysfunctional [136]. Similar observations were made in chronic lymphocytic leukemia (CLL) patients, in which the total number of ILC1s was increased, while the percentage of TNF-α-producing ILC1s was decreased [137]. In mouse models of cancer, Gao et al. (2017) described a population of ‘intermediary’ ILC1s that shared some characteristics of both NK cells and non-NK ILC1s. These intermediate cells were suggested to contribute to tumor progression [34]. These cells expressed both CD49a and CD49b and were dependent on TGF-β signaling. Although the presence of these cells was associated with a poorer disease outcome, their functions and developmental origins are not fully understood. Nevertheless, these studies demonstrate that the protumor and antitumor functions of ILC1s are highly context dependent and can change with progressing disease.

### ILC2s

ILC2s can have both favorable and detrimental effects in the context of cancer. In the lung, while their presence within a primary lung tumor may favor antitumor responses because of their capacity to produce cytokines and recruit eosinophils, ILC2s can remodel the lung tissue to allow seeding and growth of metastases. Intranasal treatment with IL-33 was shown to act on ILC2s to suppress NK cell function, resulting in an increased metastatic burden in a mouse model of metastases [138]. Activated ILC2s indirectly deprive NK cells of metabolites, thereby inhibiting their antitumor function [138]. Mechanistically, IL-33 induced IL-5 secretion by ILC2s, which activated eosinophils. These activated eosinophils engage glycolytic metabolism, thereby reducing the availability of glucose in the tissue environment. NK cells rely on glucose to produce cytokines and cytotoxic molecules, and a lack of glucose results in impaired effector function [138]. In human patients, ILC2s were found to be enriched in lung cancer tissue. In this setting, they expressed high levels of PD-1 and the cytokines IL-4 and IL-13. In vitro, the supernatant from cultured PD-1^high^ ILC2s was sufficient to induce an immunoregulatory phenotype in cultured macrophages. These cultured macrophages expressed high levels of *TGFB1*, *CCL18* and *ARG1*, suggesting that cytokines produced by ILC2s contribute to the immunosuppressive microenvironment by acting on macrophages to produce immunosuppressive mediators that dampen the antitumor response [139]. In contrast, mice lacking ILC2s have been shown to be more susceptible to primary and metastatic lung tumors, as well as a mouse model of colorectal cancer [140, 141], suggesting that ILC2s can also have a protective function. Indeed, a type 2 immune cell signature, which includes ILC2s and T_H_2 cells in metastatic melanoma tumors, correlated with improved survival. These findings were corroborated in a mouse model of melanoma in which ILC2s that produced GM-CSF were able to recruit eosinophils to limit tumor growth. This protumor function was curtailed by PD-1 expression on ILC2s, which limited the ability of ILC2s to self-renew in the tumor microenvironment [7]. Wang et al. (2020) showed that several subsets of ILC2s with distinct functions exist within tumors in mouse models of colon cancer. The researchers identified a PD-1^high^ population that promoted tumor progression in immunodeficient mice and a PD-1^low^ population that had no effect on tumor growth [8]. Thus, it appears that PD-1-expressing ILC2s found in cancer foster tumor progression, while PD-1^low^ ILC2s affect tumor progression by orchestrating the recruitment of immune cells such as eosinophils and T cells [7, 142].

### ILC3s

In the context of cancer, ILC3s have been studied almost extensively in colorectal cancer, in which they can have protumor and antitumor functions. ILC3s can promote inflammation and tumorigenesis by secreting IL-17A and IL-22 in response to IL-23 [143, 144]. Systemic expression of IL-23 was shown to be sufficient to drive tumorigenesis by inducing IL-17 production in ILC3s [145]. In contrast to this pro-inflammatory protumor action, a different study showed that antigen-presenting MHC-II^+^ ILC3s limit the progression and invasion of colorectal cancer by supporting the maintenance of antitumor T_H_1 cells [146]. Fecal microbial transplants of mice lacking MHC-II on ILC3s induced loss of T_H_1 cells, suggesting that ILC3s indirectly maintain T_H_1 cells by controlling the microbiota to support the presence of antitumour T cells [146]. In lung neoplasia, NCR^+^ ILC3s accumulate specifically in intratumoral tertiary lymphoid structures, the presence of which was associated with better survival [147]. The mechanisms by which these ILC3s act are unclear, but their presence on the edge of the tertiary lymphoid structures suggests that they contribute to the formation or maintenance of these structures. Additionally, in vitro experiments show that NCR^+^ ILC3s can directly recognize cancer cells through the activating receptor NKp44, resulting in the production of TNF-α and IL-8, which in turn induce recruitment and proliferation of immune cells [147]. Using models of transplantable tumors, a recent study showed that ILC3s can orchestrate the adaptive immune response following chemotherapy [148]. Chemotherapy induced expression of CCL20, which acted on CCR6-expressing ILC3s to secrete the chemokine CXCL10. This promoted the recruitment of T cells to the tumor, leading to an improved response [148].

A number of studies have reported the presence of ILC1s that have a history of expressing RORγt, suggesting that the tumor microenvironment can induce ILC3-to-ILC1 conversion. Using lineage tracing, Wang et al. (2020) reported a population of ILC1s known as “ex-ILC3s” that had expressed RORγt and normally produced IL-22 but then switched to be IL-10-producing cells in late-stage colorectal tumors. This switch was dependent on TGF-β, suggesting that the cytokine *milieu* within the tumor microenvironment influences immune cell function [8]. By comparing the transcriptional landscape of ILC3s isolated from tumors and healthy adjacent tissue of colorectal cancer patients, Goc et al. (2021) showed that intratumoral ILC3s upregulated several ILC1-related genes, including *PRF1, GZMA, GZMB* and *GZMK*, while simultaneously downregulating ILC3-related genes, such *as IL22* and *CCL20* [146]. A population of cytotoxic ILC3s characterized by the expression of CD127 and CD94 was recently identified in human tonsils [149]. These cells developed from ILC3s when cultured with IL-2, IL-12 and IL-1β, were transcriptionally similar to NCR^+^ ILC3s and expressed RORγt. In Crohn’s disease patients, a similar subset of CD127^+^CD94^+^ ILCs was found to be cytotoxic, but the RORγt^−^ cells may have previously expressed RORγt but lost expression as a result of the inflammatory environment [149, 150]. These studies suggest that specific changes in the tissue environment, such as inflammation and cancer, can lead to gradual ILC3-to-ILC1 conversion [146, 149, 150].

The phenotype of ILCs associated with protumor or antitumor behavior within a tumor appears to be dictated by the dynamic complexity of the cytokine *milieu*. ILC dysfunction and plasticity emerge over time, resemble the changes that occur in T cells in response to chronic stimuli and slowly change the transcriptional program, rendering ILCs less effective in the antitumor response. Despite the relative rarity of ILCs in tumors, it is clear that they play a key role in the recruitment and function of other immune cells in the tumor microenvironment. Thus, even small changes within the ILC compartment can have a significant impact on the antitumor response, and targeting ILCs using immunotherapy is likely to have a magnified effect due to their ability to influence the function of other immune cells.

## Inflammation heightens the risk of liver cancer

Chronic inflammation has been recognized as a hallmark of cancer [151], and HCC often arises as a consequence of persistent inflammation [152, 153]. This chronic low-grade inflammation has been shown to lead to remodeling of the liver microenvironment. This includes altering the prevalence and function of immune cells and can eventually lead to immune cell dysfunction and tissue damage. In the liver, inflammation can be caused by chronic viral liver infection, alcohol abuse or metabolic-associated fatty liver disease (MAFLD). Together, these factors form the main risk factors for the development of HCC [153]. ILCs have been an intense focus of investigation in disease and immune protection over the past decade, but they have not been studied in detail in the context of liver disease [154] despite the fact that studies placing ILCs in a central role in liver-related diseases are now emerging. In addition to their role as innate immune cells that play a crucial role in the acute immune response, ILCs can be involved in chronic inflammatory processes. In the liver, unresolved chronic inflammation can lead to remodeling of ILCs and functional changes, impacting their ability to respond to acute stimuli.

## The protective role of group 1 ILCs at early stages of liver disease

**Fig. 3:**
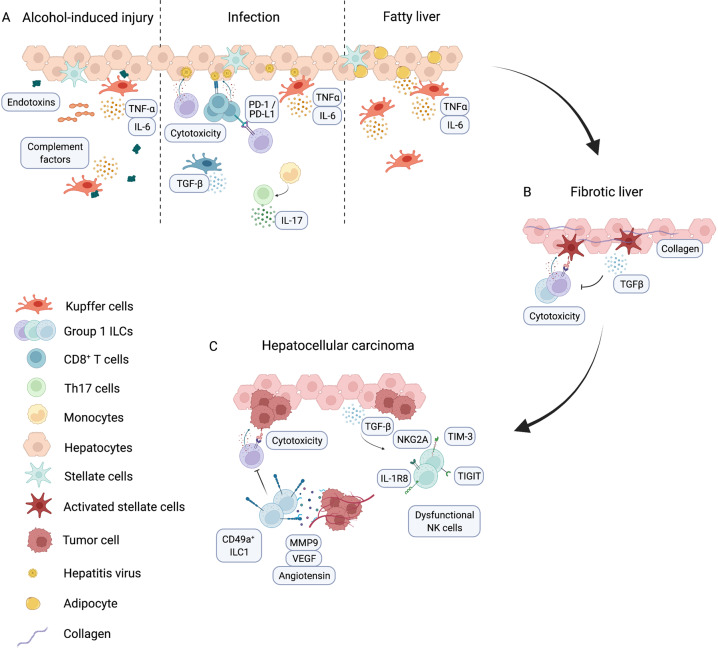
Immune cell function in liver pathologies. **A** Liver cancer risk factors include alcohol-induced liver injury, which is associated with an increase in endotoxins in the liver that activate Kupffer cells to produce TNF-α and IL-6. This results in increased production of complement factors that drive an inflammatory environment. In pathogen infection, group 1 ILCs and cytotoxic T cells can recognize and eliminate infected hepatocytes. ILC1s can express PD-L1 in settings of chronic infection, which negatively regulates adaptive immune responses. Kupffer cells can be both proinflammatory and anti-inflammatory. Proinflammatory Kupffer cells produce TNF-α, and anti-inflammatory Kupffer cells produce TGF-β. Monocytes can regulate T cells to produce the proinflammatory cytokine IL-17. Obesity activates a cascade in which Kupffer cells produce TNF-α and IL-6, resulting in chronic inflammation and the development of fatty liver disease. **B** Chronic inflammation can lead to liver fibrosis. NK cells and ILC1s can directly recognize and eliminate activated stellate cells in the fibrotic liver, a pathway that can be inhibited by TGF-β secretion. **C** Sustained inflammation and fibrosis can result in the development of hepatocellular carcinoma. NK cells can directly kill cancerous cells but ultimately can become dysfunctional, for example, in response to TGF-β, which is highly expressed in hepatic inflammation. These dysfunctional NK cells express receptors that negatively regulate their cytotoxicity. CD49a^+^ ILC1s can inhibit NK cell function and promote cancer growth by producing proangiogenic factors allowing tumor growth

Early stages of liver injury and fibrosis are associated with activation of hepatic stellate cells (HSCs), and functional ILC1s appear to be essential in the liver to protect against these early stages of liver injury and to prevent HSCs from transdifferentiating into cells that promote fibrosis (Fig. 3, Table 3) [36, 155]. Activated HSCs express ligands for NKG2D, NKp46 and TRAIL, marking them for recognition and killing by group 1 ILCs. ILC1s and NK cells differentially express the receptors NKG2D, NKp46 and TRAIL and thus can directly induce apoptosis in these cells, thereby ameliorating liver fibrosis [36, 155]. In addition to direct killing of HSCs, ILC1s were also shown to protect against acute liver injury by secreting IFN-γ, which promoted increased survival of hepatocytes (Table 3) [156]. Mechanistically, activation of DNAX accessory molecule 1 (DNAM1, also known as CD226) on ILC1s induced production of IFN-γ, and this was amplified by the presence of extracellular adenosine triphosphate (ATP), a hallmark of liver injury, and IL-12, produced by myeloid cells. IFΝ-γ suppressed the activation of HSCs and promoted the survival of hepatocytes by inducing the Bcl-1 protein family member Bcl-xL [156]. In advanced stages of disease, HSCs produce TGF-β, which inhibits group 1 ILCs and impacts their ability to eliminate these profibrotic cells [157]. Paralleling an increase in TGF-β production as liver disease progresses in human patients, the frequency of ILC1s gradually decreases (Table 3) [158]. Thus, while group 1 ILCs can protect against fibrosis at early stages of disease, disease progression is associated with changes in the cytokine *milieu* that inhibit the function of NK cells and ILC1s.

**Table 3:** Liver diseases, associated changes in the immune environment and impacts on group 1 ILCs

Disease	Changes in the immune environment	Impact on NK cells	Impacts on ILC1s	Ref.
Obesity	• Increased TNF-α and IL-6 production • NF-κB activation • Migration of DCs to activate T cells and NK cells • Increased stiffness of the liver	• Reduced cytotoxicity • Inhibited glycolysis • reduced effector function • ILC1-like phenotype	• Frequency unchanged • Changes in function unknown	[42, 160, 161]
Alcohol abuse	• Increased TNF-α and IL-6 production • NF-κB activation	• Unknown, likely reduced cytotoxicity as seen in obesity	• Unknown, likely reduced cytotoxicity as seen in obesity	[164–166]
Viral infection	• Immune response to induce viral clearance • Immune suppression to dampen tissue-damaging inflammation	• Increased expression of NKG2D expression, increased cytotoxicity, and increased IFN-γ production	• Upregulation of PD-L1 expression by ILC1s, which negatively regulates antiviral T cells	[158, 159]
Liver injury and fibrosis	• ATP release • Increase in IL-12 expression • Upregulation of NK cell/ILC1 ligands on activated hepatic stellate cells	• Elimination of activated hepatic stellate cells	• Elimination of activated hepatic stellate cells • Protective IFN-γ production	[36, 155, 156]
Hepatocellular carcinoma	• Expression of NK cell receptors on tumor cells • (Late stages) increased production of inhibitory cytokines (IL-10, TGF-β)	• Antitumor function at early stages • Upregulation of inhibitory receptors • NK cell dysfunction	• Expression of inhibitory receptors • Production of pro-angiogenic factors and protumor molecules	[173–175, 179]
Liver metastasis	• TGF-β and IL-15 expression • Immune environment permissive for metastatic cells	• Control of metastatic outgrowth • IL-15-dependent effects • Reduced killing capabilities at advanced stages	• Control of metastatic seeding • Failure to infiltrate tumors • Sustained function at advanced stages • IFN-γ and granzyme B production	[111, 112]

## How does the inflammatory environment impact ILC function?

Several risk factors lead to fibrosis and heighten the susceptibility to liver cancer. One of these risk factors is obesity, which is associated with major changes in the function of adipocytes. In lean individuals, adipocytes secrete anti-inflammatory mediators, such as IL-10 and adiponectin, whereas obesity leads to a proinflammatory signature featuring the release of TNF-α and IL-6 [159], which can directly modulate ILC function. Systemic overexpression of IL-6 in mice reduced the cytotoxicity of splenic NK cells by reducing the levels of perforin and granzyme. Similarly, IL-6 treatment of human peripheral NK cells negatively affected their cytotoxicity [160], suggesting that excessive production of IL-6 in response to liver injury negatively regulates NK cells (Table 3). Furthermore, lipid uptake by group 1 ILCs can directly impact their function. In obese patients, fatty acids can no longer be stored in adipocytes and are instead transported to the liver where they accumulate. This directly impacts group 1 ILC function by upregulating the peroxisome proliferator-activated receptor (PPAR) pathway and inhibiting mammalian target of rapamycin (mTOR)-mediated glycolysis, leading to reduced production of effector molecules (Table 3) [161]. Whether this is mediated by NK cells, ILC1s or both is not known. A subsequent study assessed the roles of NK cells and ILC1s in obesity and showed that obesity impaired NK cell function and induced a shift to an ILC1-like phenotype [42]. In addition to this direct modulation of group 1 ILCs, accumulation of free fatty acids can cause hepatic steatosis, which leads to increased stiffness of the liver and stimulates the nuclear factor kappa B (NF-κB) pathway in liver-resident macrophages (Kupffer cells). Subsequent release of proinflammatory cytokines establishes a chronic inflammatory response that impacts the function of other liver-resident immune cells and ultimately leads to tissue damage (Fig. 3) [162]. Beyond liver-resident cells, obesity and the associated inflammatory *milieu* can activate dendritic cells (DCs) to migrate out of the liver to lymph nodes, where they can activate other immune cells, such as T cells and NK cells [163]. This induces a self-reinforcing destructive circuit to establish liver damage, which directly impacts ILC functionality (Table 3). Similar mechanisms are in place in alcohol-induced liver injury. Due to impaired barrier integrity in the intestine in response to chronic alcohol intake, increased transfer of gut toxins and endotoxins are transported to the liver through the portal vein (Fig. 3). Kupffer cells within the liver parenchyma are activated via the Toll-like receptor (TLR)-4-NF-κΒ pathway to initiate inflammation (Fig. 3). This pathway is similar to that seen in obesity, including increased production of TNF-α and IL-6. This is in addition to the production of complement factors, which leads to both local and systemic inflammation [164–166]. How these mechanisms modulate ILC function is not fully understood (Table 3).

## Group 1 ILCs in viral infections – protective or destructive?

In alcohol- or obesity-induced liver injury, the inflammatory response is initiated in the absence of a pathogen. This contrasts with the host-protective immune response that is essential in viral infections. Acute infection with hepatitis C virus (HCV) leads to increased expression of NKG2D on NK cells as well as increased cytotoxicity and IFN-γ production, suggesting that NK cells are part of the antiviral immune response (Fig. 3, Table 3) [167]. In addition to direct elimination of infected cells, group 1 ILCs can modulate T cell responses. In a model of viral infection using lymphocytic choriomeningitis (LCMV), hepatic ILC1s, but not NK cells, were shown to express PD-L1 in the healthy liver (Fig. 3, Table 3). In response to viral infection, ILC1s were able to proliferate and upregulate PD-L1 and thus could negatively regulate PD-1-expressing T cells to limit their proliferation and cytokine secretion [168]. This mechanism may dampen tissue-damaging inflammation but at the same time delay viral clearance. Similarly, group 1 ILCs were shown to limit T-cell-mediated liver pathology by competing for IL-2 availability in the local liver environment in a mouse model of HBV infection (Table 3) [169]. Whether these mechanisms play a role in human hepatitis infections is unclear. Failure of the early immune response results in impaired viral clearance, resulting in the establishment of a chronic infection such as that seen in hepatitis virus infection. This unresolved low-grade infection results in chronic activation of immune cells and cytokine secretion by macrophages and other immune cells to induce liver fibrosis (Fig. 3).

This inflammatory hepatic environment sets the scene for heightened susceptibility to HCC through three main mechanisms: (i) Induction of dysfunctional immune cells such as T cells and NK cells that develop hallmarks of exhausted cells due to chronic antigen exposure (e.g., as in chronic hepatitis [170, 171]) or a cytokine *milieu* that downmodulates cytotoxicity; (ii) remodeling of the liver environment that results from deposition of extracellular matrix leads to fibrosis and results in increased liver stiffness, angiogenesis and survival of cancerous cells;. and (iii) chronic tissue damage that results in secondary increased DNA damage and increased potential for the development of hepatic neoplasia.

## ILCs in the context of liver cancer and liver metastases

Hepatic NK cells and ILC1s are critical in maintaining homeostasis in the liver. When this surveillance system fails, malignant cells are no longer recognized and eliminated, and the susceptibility to liver cancer or metastatic seeding is heightened. Due to the abundance of group 1 ILCs in the healthy liver and their critical role in mediating antitumor immunity, understanding how ILCs contribute to HCC and liver metastases can aid the development of new therapeutic approaches based on ILCs.

In line with other types of cancers, in one study, the presence of CD56^+^ NK cells within tumors of HCC patients correlated with improved survival, suggesting that NK cells contribute to the antitumor response by eliminating cancerous cells and thereby limiting tumor growth (Table 3) [172]. Notably, the study only included patients with early stages of disease (stage I and II) and resectable tumors, limiting the findings to a subset of patients with high survival rates. In contrast, the presence of a particular subtype of CD11b^−^CD27^−^ “immature” NK cells correlated with tumor progression. These NK cells expressed lower levels of IFN-γ and the degranulation marker CD107a than healthy controls, suggesting reduced cytotoxicity and impaired antitumor function (Table 3) [173]. High expression of the inhibitory receptor NKG2A on tumor-infiltrating NK cells and its ligand human leukocyte antigen E (HLA-E) on the surface of tumor cells was correlated with reduced cytotoxicity and disease progression (Fig. 3) [130]. Similarly, high expression of inhibitory receptors on NK cells, including CD96 [174], T cell immunoreceptor with Ig and ITIM domains (TIGIT) and TIM-3 [175], negatively correlated with patient survival. Stimulation of healthy blood NK cells with IL-10 and TGF-β upregulated NKG2A and CD96 expression, respectively, suggesting that cytokines dictate receptor expression and consequently the function of NK cells [130, 174]. The prognostic value of tumor-infiltrating NK cells in HCC is thus highly dependent on their phenotype and function. Understanding what mechanisms lead to NK cell dysfunction and whether it can be reversed can help in identifying new therapeutic targets. Using a mouse model of HCC, IL-1R8 was identified as a negative regulator on NK cells and a potential therapeutic target. Genetic deletion of *Il1r8* protected against severe liver disease by increasing NK cell infiltration and function [176]. Mechanistically, IL-1R8 regulates NK cells by blocking signal transduction of the proinflammatory cytokine IL-1 [177]. Additionally, IL-1R8 is involved in the signaling pathway of the anti-inflammatory cytokine IL-37 [178]. IL-1R8 is highly expressed on NK cells, where it negatively regulates the expression of the activating receptors DNAM-1, NKG2D and Ly49H and the effector molecules IFN-γ, granzyme B and FasL [176]. The mechanisms that lead to the activation of IL-1R8 and NK cell inhibition remain unclear and will be important for understanding how this pathway could be targeted.

In HCC, similar to other cancers, little is known about the role of ILC1s because most studies have not distinguished the actions of ILC1s from those of NK cells. The expression of CD49a marks a subset of ILC1s in the human liver, and this marker has been shown to be associated with exacerbated disease in HCC patients. These ILC1s accumulated within the tumor tissue, where their enrichment was correlated with the heightened expression of inhibitory receptors, such as PD-1, CD200R, NKG2A and B and T lymphocyte attenuator (BTLA), and reduced overall and disease-free survival (Table 3) [53]. Phenotypic and functional characterization of tumor-infiltrating ILC1s revealed a decrease in CXCR6^+^ ILC1s and an increase in CD49a^+^ ILC1s within tumor compared to nontumor tissue and healthy controls [179]. The CD49a^+^ ILC1 subset expressed high levels of EOMES, which is in contrast with previous studies showing that human hepatic CD49a^+^ ILC1s lack EOMES expression [48, 179]. CD49a^+^ ILC1s produced proangiogenic factors, such as vascular endothelial growth factor (VEGF), matrix metalloproteinase (MMP)-9 and angiogenin (Fig. 3, Table 3). Their presence was negatively correlated with the frequency of CD107a^+^ NK cells, suggesting that CD49a^+^ ILC1s can contribute to disease progression by promoting angiogenesis and modulating NK cell function (Fig. 3) [179].

**Fig. 4:**
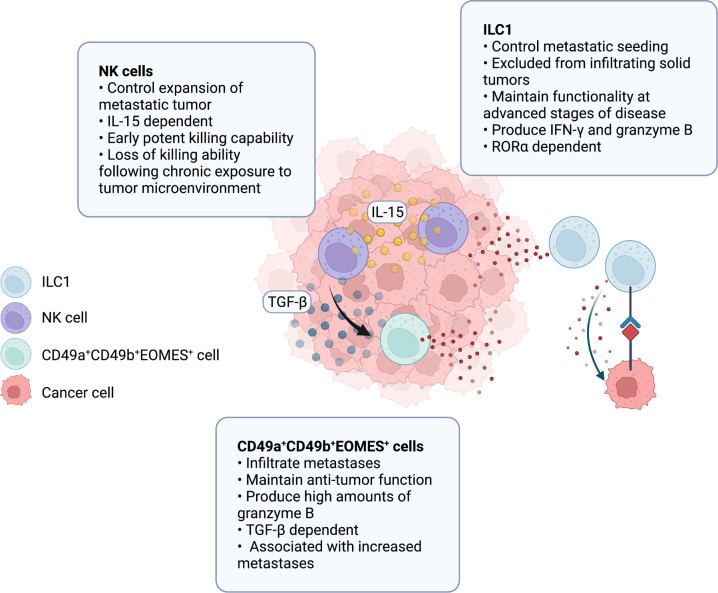
The function of group 1 ILCs in experimental liver metastases in mice. NK cells depend on IL-15 for their activation and exhibit immunosurveillance and cytotoxicity that allow them to actively drive antitumor functions. Although NK cells can eliminate tumor cells to control potential tumor metastasis, they can become dysfunctional within the cytokine *milieu* of the tumor microenvironment, which results in reduced killing of abnormal cells compared with naïve NK cells. In contrast to NK cells, ILC1s do not appear to readily infiltrate metastatic tumors to directly eliminate them but can control metastatic seeding by producing granzyme B and IFN-γ. The fact that they do not penetrate within the tumor spatially positions them distant from the immunosuppressive environment of the tumor, allowing them to retain their antimetastatic functions. Their development and antitumor functions rely on their expression of the transcription factor RORα. A recently described intermediate ILC1/NK cell type, CD49a^+^CD49b^+^Eomes^+^ cells, are present within nodules and produce high levels of granzyme B [94]. Although their role in tumors has not yet been fully elucidated, mice deficient in TGF-β signaling fail to develop CD49a^+^CD49b^+^Eomes^+^ cells and present with fewer metastases, suggesting a protumor function of these cells despite their high production of granzyme B

The liver is a common site of metastases due to its central location and the high volume of blood that flows through it, which can contain metastatic cells from distant sites. Group 1 ILCs, in particular NK cells, are critical in recognizing and eliminating malignant cells to prevent cancer growth at secondary sites [180]. NK cell dysfunction, caused by chronic liver disease, can thus make the liver a permissive environment for metastatic cells. The most common primary cancer to metastasize to the liver is colorectal cancer [181], which can be modeled in mice by intrasplenic injection of colorectal cancer cells. The first evidence for a protective role for ILC1s in liver metastasis came from a study in which liver metastases were induced and shown to be enhanced when mice were treated with an anti-TRAIL antibody, which specifically inhibits ILC1s [182]. Further expanding on this finding, two studies using models of colorectal cancer liver metastases provided evidence for the importance of group 1 ILC1s in inhibiting metastatic seeding and outgrowth. Ducimetière et al. (2021) studied the function of both NK cells and ILC1s by using NK-cell-deficient *Ncr1*^*Cre*^*Eomes*^*fl/fl*^ mice and *Hobit*^*−/−*^ mice lacking ILC1s in the liver. By depleting NK cells in ILC1-deficient mice and all *Ncr1*-expressing cells in NK-cell-deficient mice at various timepoints before and after the injection of tumor cells, the authors showed that ILC1s were particularly important in controlling metastatic seeding, whereas NK cells controlled outgrowth (Fig. 4, Table 3) [112]. Using fluorescence microscopy, the researchers further showed that NKp46-expressing cells readily infiltrated tumors in *Hobit*^*−/−*^ mice but not *Ncr1*^*Cre*^*Eomes*^*fl/fl*^ mice, suggesting that NK cells, but not ILC1s, are able to infiltrate the nodules. The nodules contained a population of CD49a^+^CD49b^+^EOMES^+^ cells, which produced granzyme B and IFN-γ. These cells that expressed phenotypic markers associated with NK cells and ILC1s were dependent on TGFβ signaling (Fig. 4) [112], similar to the subset of ‘intermediate’ ILC1s identified by Gao et al. (2017) [34]. These cells were present in *Hobit*^−/−^ mice, suggesting that they originate from NK cells rather than ILC1s, but the exact function of these cells remains unclear [112]. This is in contrast to a recent study, in which the origin of CD49a^+^CD49b^+^EOMES^+^ cells was studied in more detail. Using a model of breast cancer, the authors showed that the subset of EOMES-expressing ILC1s had no history of *S1pr5* expression, a marker used to distinguish NK cells from ILC1s, suggesting that this population is ontogenically distinct from NK cells [66]. Whether these findings apply to the populations identified by Gao et al. (2017) [34] and Ducimetière et al. (2021) [112] remains unknown. A second study using the same model of liver metastases showed that the transcription factor RORα is critical for the control of metastases (Fig. 4). Mice lacking RORα on *Ncr1*-expressing cells had reduced numbers of ILC1s in the liver and failed to control liver metastases compared to wild-type mice [111], suggesting a protective role for ILC1s. In a study using a model of lung cancer liver metastasis, Correia et al. demonstrated the importance of NK cells in suppressing liver metastases [183]. The authors showed that exit from dormancy and metastatic outgrowth were accompanied by a reduction in IFN-γ-producing NK cells, suggesting that a decrease in NK cells allows tumor cells to proliferate. Mechanistically, activated stellate cells secreted CXCL12, which inhibited CXCR4-expressing NK cells [183]. In this study, the authors did not distinguish between NK cells and ILC1s, and thus, a protective role of ILC1s is plausible.

## Concluding remarks

Hepatic group 1 ILCs play a role in a number of liver pathologies, including cancer. NK cells typically control infected or malignant cells and are thus beneficial in settings of viral infection, fibrosis or early stages of cancer. However, in some settings, these NK cells can become dysfunctional and lose their ability to eliminate cancerous cells. Despite recent advances in understanding their role in liver homeostasis and pathology, it remains unclear how the local environment can reshape NK cell function to limit the antitumor response. Chronic inflammatory disorders, such as MAFLD, chronic infection or alcohol abuse, can remodel the liver into a permissive environment for cancer cells. One of the cytokines involved in NK cell dysfunction is TGF-β, which dampens NK cell effector functions in fibrosis and cancer. Determining what other factors contribute to this dysfunction and how it can be targeted therapeutically is important for the development of new therapies. The role of ILC1s is less well understood, and their function appears to be highly context dependent. ILC1s can control metastatic seeding of colorectal cancer metastases but can also contribute to disease progression by inhibiting NK cells and secreting protumorigenic molecules. Thus, understanding what cues regulate ILC1s in the liver is of particular importance. Lastly, questions about the identity and plasticity of NK cells and ILC1s need to be answered. NK cells can upregulate markers associated with ILC1s in the context of cancer, but how these cells contribute to disease is unclear. Their presence is associated with disease progression, suggesting that they are dysfunctional and/or actively contribute to tumor growth. Thus, strategies aiming to restore the antitumor function of these cells are of interest.
